# Consensus report from the 10th Global Forum for Liver Magnetic Resonance Imaging: developments in HCC management

**DOI:** 10.1007/s00330-023-09928-y

**Published:** 2023-07-27

**Authors:** Bachir Taouli, Ahmed Ba-Ssalamah, Julius Chapiro, Jagpreet Chhatwal, Kathryn Fowler, Tae Wook Kang, Gesine Knobloch, Dow-Mu Koh, Masatoshi Kudo, Jeong Min Lee, Takamichi Murakami, David J. Pinato, Kristina I. Ringe, Bin Song, Parissa Tabrizian, Jin Wang, Jeong Hee Yoon, Mengsu Zeng, Jian Zhou, Valérie Vilgrain

**Affiliations:** 1 Department of Diagnostic, Molecular, and Interventional Radiology, Icahn School of Medicine at Mount Sinai, New York, NY USA; 2 BioMedical Engineering and Imaging Institute, Icahn School of Medicine at Mount Sinai, New York, NY USA; 3 Department of Biomedical Imaging and Image-guided therapy, Medical University of Vienna, Vienna, Austria; 4 Department of Radiology and Biomedical Imaging, Yale School of Medicine, New Haven, CT USA; 5 Department of Radiology, Institute for Technology Assessment, Massachusetts General Hospital, Harvard Medical School, Boston, MA USA; 6 Department of Radiology, University of California San Diego, La Jolla, CA USA; 7 Department of Radiology and Center for Imaging Science, Samsung Medical Center, Sungkyunkwan University School of Medicine, Seoul, South Korea; 8 Global Medical and Clinical Affairs and Digital Development, Radiology, Bayer Pharmaceuticals, Berlin, Germany; 9 Department of Diagnostic Radiology, Royal Marsden Hospital, Sutton, UK; 10 Department of Gastroenterology and Hepatology, Kindai University Faculty of Medicine, Osaka, Japan; 11 Department of Radiology, Seoul National University Hospital and Seoul National University College of Medicine, Seoul, South Korea; 12 Department of Radiology, Kobe University Graduate School of Medicine, Kobe, Japan; 13 Department of Surgery & Cancer, Imperial College London, Hammersmith Hospital, London, UK; 14 Division of Oncology, Department of Translational Medicine, University of Piemonte Orientale, Novara, Italy; 15 Department of Diagnostic and Interventional Radiology, Hannover Medical School, Hannover, Germany; 16 Department of Radiology, West China Hospital, Sichuan University, Chengdu, People’s Republic of China; 17 Recanati/Miller Transplantation Institute, Icahn School of Medicine at Mount Sinai, New York, NY USA; 18 Department of Radiology, Third Affiliated Hospital of Sun Yat-sen University, Guangzhou, People’s Republic of China; 19 Liver Disease Hospital, Sun Yat-sen University, Guangzhou, People’s Republic of China; 20 Department of Radiology, Zhongshan Hospital, Fudan University, Shanghai, People’s Republic of China; 21 Liver Cancer Institute, Zhongshan Hospital, Fudan University, Shanghai, People’s Republic of China; 22 Université Paris Cité and Department of Radiology, Assistance-Publique Hôpitaux de Paris, APHP Nord, Hôpital Beaujon, Clichy, France

**Keywords:** Gadoxetic acid, Hepatocellular carcinoma, Magnetic resonance imaging

## Abstract

**Abstract:**

The 10th Global Forum for Liver Magnetic Resonance Imaging (MRI) was held as a virtual 2-day meeting in October 2021, attended by delegates from North and South America, Asia, Australia, and Europe. Most delegates were radiologists with experience in liver MRI, with representation also from specialists in liver surgery, oncology, and hepatology.

Presentations, discussions, and working groups at the Forum focused on the following themes:

• Gadoxetic acid in clinical practice: Eastern and Western perspectives on current uses and challenges in hepatocellular carcinoma (HCC) screening/surveillance, diagnosis, and management

• Economics and outcomes of HCC imaging

• Radiomics, artificial intelligence (AI) and deep learning (DL) applications of MRI in HCC.

These themes are the subject of the current manuscript. A second manuscript discusses multidisciplinary tumor board perspectives: how to approach early-, mid-, and late-stage HCC management from the perspectives of a liver surgeon, interventional radiologist, and oncologist (Taouli et al, 2023).

Delegates voted on consensus statements that were developed by working groups on these meeting themes. A consensus was considered to be reached if at least 80% of the voting delegates agreed on the statements.

**Clinical relevance statement:**

This review highlights the clinical applications of gadoxetic acid–enhanced MRI for liver cancer screening and diagnosis, as well as its cost-effectiveness and the applications of radiomics and AI in patients with liver cancer.

**Key Points:**

*• Interpretation of gadoxetic acid–enhanced MRI differs slightly between Eastern and Western guidelines, reflecting different regional requirements for sensitivity vs specificity.*

*• Emerging data are encouraging for the cost-effectiveness of gadoxetic acid–enhanced MRI in HCC screening and diagnosis, but more studies are required.*

*• Radiomics and artificial intelligence are likely, in the future, to contribute to the detection, staging, assessment of treatment response and prediction of prognosis of HCC—reducing the burden on radiologists and other specialists and supporting timely and targeted treatment for patients.*

**Supplementary Information:**

The online version contains supplementary material available at 10.1007/s00330-023-09928-y.

## Gadoxetic acid in clinical practice: Eastern and Western perspectives on current uses and challenges

### Eastern guidelines overview

Eastern guidelines on HCC screening, diagnosis, and management include the Asia-Pacific Association for the Study of the Liver (APASL 2017) [1], the Korean Liver Cancer Association-National Cancer Center Korea (KLCA-NCC 2018) [2], the Japan Society of Hepatology (JSH 2021) [3], and the China Liver Cancer national guidelines (CNLC 2019) staging system guidelines [4, 5].

#### HCC screening and surveillance

All Eastern guidelines recommend a 6-monthly US as the first-line imaging modality for HCC screening/surveillance in high-risk groups [1–4], with additional recommendations on serum alfa-fetoprotein (AFP) measurement in the APASL, KLCA-NCC, and CNLC guidelines [1, 2, 4].

#### HCC diagnosis

Eastern guideline recommendations on first-line imaging modalities for nodules ≥ 1 cm can be summarized as follows:

- APASL: dynamic CT, or dynamic MRI using extracellular contrast media (ECCM), or gadoxetic acid–enhanced MRI [1]
- KLCA-NCC: multiphase CT, or multiphase MRI using ECCM-MRI, or gadoxetic acid–enhanced MRI [2]
- JSH: gadoxetic acid–enhanced MRI or dynamic CT [3]
- CNLC: dynamic CT, or dynamic MRI using ECCM, or gadoxetic acid–enhanced MRI, or contrast-enhanced US [4, 5].

In KLCA-NCC, JSH, and CNLC guidelines, the imaging modalities are considered equally suitable for HCC diagnosis, while the APASL guidelines recommend gadoxetic acid–enhanced MRI over ECCM-MRI. The criteria for HCC diagnosis using gadoxetic acid–enhanced MRI are broadly similar across Eastern guidelines (Table 1).

**Table 1:** Diagnostic criteria for HCC using gadoxetic acid–enhanced MRI in Eastern guidelines

APASL guidelines [1]	KLCA-NCC guidelines [2]	JSH guidelines [3]	CNLC guidelines [4, 5]
• APHE + washout (PVP or TP) or HBP hypoenhancement • Exclusion criteria: hemangioma by T2WI • No size limit for nodules showing APHE and washout • Allow inter-exam combination of major imaging features • Allow diagnosis of hypovascular HCC: HBP hypointensity and defect in the Kupffer phase of CEUS	• APHE + washout in PVP, TP, or HBP • Exclusion criteria: marked T2 hyperintensity or targetoid appearance on DWI or contrast-enhanced sequences • “Probable” HCC: applying ancillary imaging features • Indeterminate nodules	• APHE + washout (PVP or TP) or HBP hypoenhancement • Exclusion criteria: hemangioma by T2WI or DWI • No size limit for nodules showing APHE and washout • Hypovascular nodules that are hypointense on HBP imaging > 1 cm in the HCC high-risk group: likely early HCC and should undergo biopsy	• APHE + washout (PVP). Frequently HBP hypoenhancement • Typical imaging features on one diagnostic test are required for HCC diagnosis of nodules > 2 cm, and typical manifestations on two diagnostic tests are required for nodules ≤ 2 cm

*Abbreviations: APASL*, Asia-Pacific Association for the Study of the Liver; *APHE*, arterial phase hyperenhancement; *CEUS*, contrast-enhanced ultrasound; *CNLC*, Chinese National Guidelines; *DWI*, diffusion-weighted imaging; *HBP*, hepatobiliary phase; *HCC*, hepatocellular carcinoma; *JSH*, Japan Society of Hepatology; *KLCA-NCC,* Korean Liver Cancer Association-National Cancer Center Korea; *MRI*, magnetic resonance imaging; *PVP*, portal venous phase; *T2WI*, T2 weighted image; *TP*, transitional phase

**Fig. 1:**
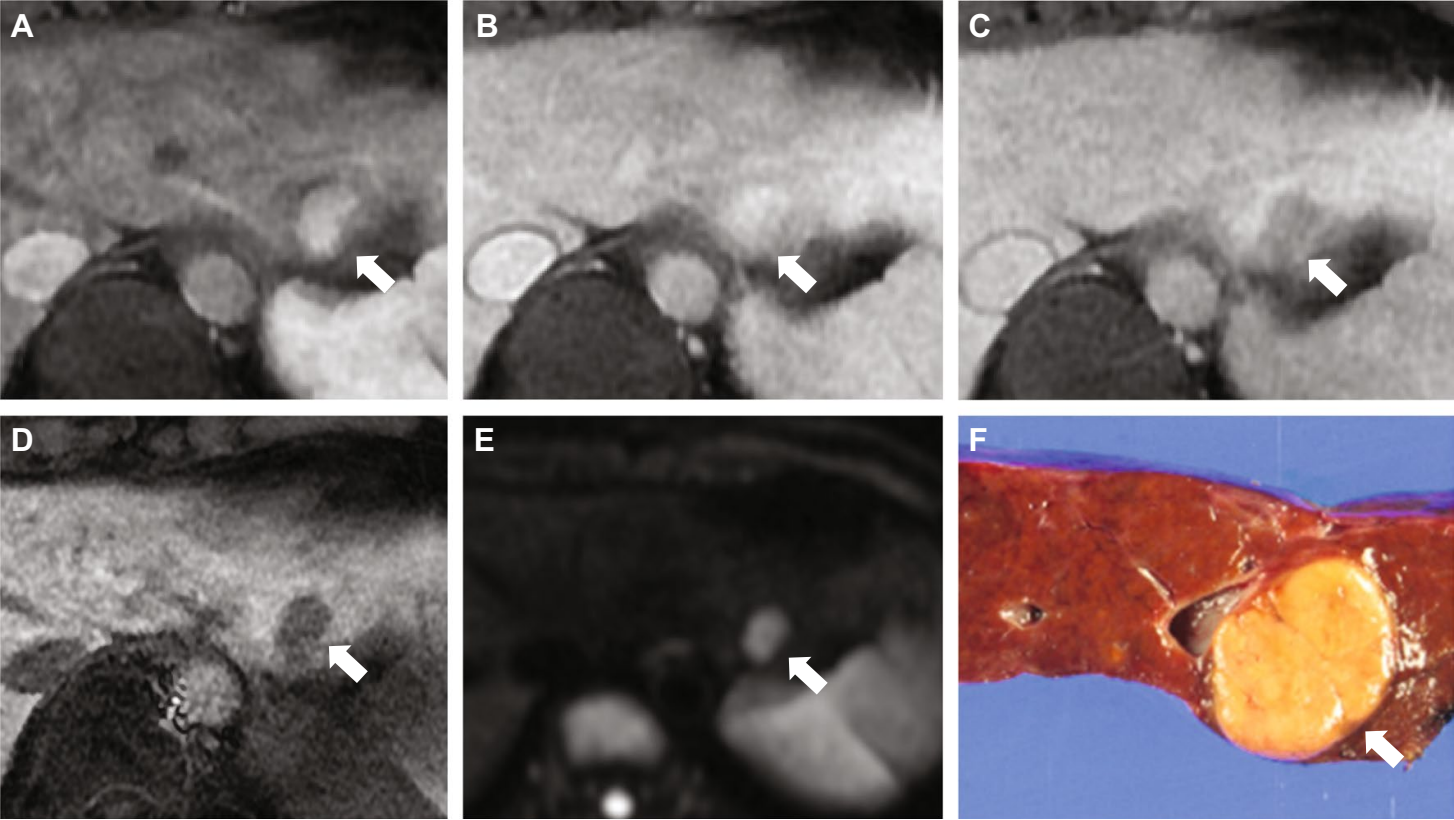
Differentiation between Eastern and Western guidelines in timing of assessment after gadoxetic acid administration. A pathology-proven HCC in a 46-year-old male patient with chronic hepatitis B. On gadoxetic acid–enhanced MRI, a nodular lesion (arrow) with non-rim APHE is seen in hepatic segment 2 (**A**) without washout on the PVP (**B**), with hypointensity on the TP (**C**) and HBP (**D**), hyperintensity on high *b*-value (*b *= 800) DWI (**E**). Gross pathology (**F**) shows a well-defined, yellow tumor, confirmed as HCC. This observation does not meet the criteria for definite HCC using LiRADS or EASL criteria, while it was diagnosed as definite HCC using Asian guidelines such as APASL or KLCA-NCC guidelines. *Abbreviations: APASL*, Asia-Pacific Association for the Study of the Liver; *APHE*, arterial phase hyperenhancement; *DWI,* diffusion-weighted imaging; *EASL,* European Association for the Study of the Liver; *HBP,* hepatobiliary phase; *HCC*, hepatocellular carcinoma*; KLCA-NCC,* Korean Liver Cancer Association-National Cancer Center Korea; *LiRADS*, Liver Imaging Reporting and Data System; *MRI*, magnetic resonance imaging; *PVP,* portal venous phase; *TP*, transitional phase. Courtesy Jeong Min Lee

A key differentiating factor between Eastern and Western guidelines is the timing of assessment after gadoxetic acid administration—i.e., whether assessing washout in the portal venous phase (PVP) or hypoenhancement in the hepatobiliary phase (HBP). Eastern guidelines on gadoxetic acid–enhanced MRI include assessment of hypoenhancement in the HBP, with the aim to optimize the sensitivity of HCC diagnosis [6] (Fig. 1). Western guidelines, by contrast (described below), restrict assessment to washout in the PVP, to enhance the specificity of HCC diagnosis. Eastern guidelines favor enhanced sensitivity for HCC diagnosis because of the widespread early use of locoregional therapies, including radiofrequency ablation (RFA) and transarterial chemoembolization (TACE) [7]. In Western practice, by contrast, patients may undergo liver transplantation based on an imaging diagnosis of HCC, and the greatest requirement here is high specificity [7].

Joo et al [6] quantified the sensitivity and specificity of gadoxetic acid–enhanced MRI based on the criteria of washout in the PVP or hypointensity in the transitional phase (TP) or HBP in a large retrospective study in 288 patients with chronic liver disease. HBP hypointensity provided high sensitivity (94%), with lower specificity (48%) when compared to PVP washout alone (98% specificity) (Table 2). Importantly, the authors showed in a later study published in 2018 [8] that including ancillary features according to the Liver Imaging Reporting and Data System (LiRADS) [9], to exclude hemangiomas and cholangiocarcinomas [10] increased the specificity of HBP imaging (to 87%) with little loss in sensitivity (93%) (Table 2).

**Table 2:** Performance of different imaging criteria on gadoxetic acid–enhanced MRI for HCC diagnosis [8]

HCC diagnostic criteria: APHE + criterion (1), (2), (3), or (4)	Sensitivity (%)	Specificity (%)
*Criterion 1:* PVP washout	70.9	97.9
*Criterion 2:* PVP washout and/or TP hypointensity	86.6	86.3
*Criterion 3:* PVP washout and/or TP and/or HBP hypointensity	93.8	48.4
*Criterion 4:* Criterion (3) + non-LiRADS 1/2/M	92.5	87.4

*Abbreviations:* *APHE*, arterial phase hyperenhancement; *HBP*, hepatobiliary phase; *HCC*, hepatocellular carcinoma; *LiRADS*, Liver Imaging Reporting and Data System; *MRI*, magnetic resonance imaging; *PVP*, portal venous phase; *TP*, transitional phase

Hwang et al [11] in 2021 performed a retrospective comparison of the sensitivity and specificity of gadoxetic acid–enhanced MRI for HCC diagnosis according to Eastern and Western guidelines in 177 patients at risk of HCC, i.e., with chronic hepatitis B or liver cirrhosis of any etiology. The imaging criteria recommended by LiRADS and the European Association for the Study of the Liver (EASL) yielded the highest specificity (95% and 94%, respectively), followed by KLCA-NCC (88%), and APASL (78%). The APASL guidelines yielded the highest sensitivity (91%), followed by KLCA-NCC (85%), LiRADS (65%), and EASL (54%). The KLCA-NCC guidelines were concluded to show the optimal balance of sensitivity and specificity [11]. The authors note the high proportion of patients with hepatitis B virus (HBV) included in their study (82%), which reflects the regional characteristics of HCC in South Korea; a study performed in Western regions, where the incidence of HCC and the proportion of patients with HBV are both lower, might provide different outcomes.

Jeon et al [12] compared the sensitivity and specificity of gadoxetic acid–enhanced MRI by Eastern and Western guideline criteria in a retrospective study of patients (*n* = 81) who were candidates for liver transplantation. The American Association for the Study of Liver Diseases (AASLD)/LiRADS guidelines had the highest specificity for HCC diagnosis (97%), followed by EASL (92%), KLCA-NCC (92%), and APASL (79%). APASL (76%) and KLCA-NCC (66%) guidelines provided higher sensitivity than the AASLD/LiRADS (35%) and EASL guidelines (39%). These authors concluded that the KLCA-NCC guidelines provide the most accurate selection of patients for transplantation [12].

#### Small HCC (< 2 cm) and micro-HCC (< 1 cm)

Eastern guidelines provide recommendations on first-line imaging modalities for diagnosing nodules ≥ 1 cm [1–4], but there are no specific algorithms for diagnosing small (< 2 cm) or micro-HCC (< 1 cm) lesions [13].

Gadoxetic acid-enhanced MRI is concluded to have the advantage over CT and ECCM-MRI of greater sensitivity in detecting early or small lesions < 2 cm, based on direct and indirect comparisons of these contrast agents in the literature [14–16]. For example, in the meta-analysis by Roberts et al [15], the sensitivity of gadoxetic acid–enhanced MRI compared to contrast CT for lesions < 2 cm was 0.76 (95% CI: 0.67–0.84) versus 0.68 (95% CI: 0.55–0.79). Gadoxetic acid-enhanced MRI was more sensitive (83.6%; 95% CI: 78.6–88.5) compared to contrast-enhanced CT (59.1%; 95% CI: 53.9–63.9) and ECCM-MRI (63.8%; 95% CI: 57.9–69.7) for lesions ≤ 2 cm in the meta-analysis by Hanna et al [16]. As a result, APASL and JSH guidelines recognize the value of gadoxetic acid–enhanced MRI for diagnosing small lesions [1, 3], while noting potential difficulties in interpretation relating to the pathologic features of early nodules**.** Applying additional diagnostic criteria to exclude “HCC mimickers”, such as non-HCC malignancies and benign lesions, further improves the ability of gadoxetic acid–enhanced MRI to detect small HCC lesions [17].

For the follow-up of patients with small HCCs after resection or local treatment, gadoxetic acid–enhanced MRI can be considered the first-choice modality to detect early recurrence [13].

#### Dysplastic nodules

Low-grade and high-grade dysplastic nodules (DNs) represent stages in the progression to early and overt HCC [18]. CT and ECCM-MRI are limited in their ability to distinguish DN stages, but HBP imaging on gadoxetic acid–enhanced MRI shows promise for distinguishing high-grade from low-grade DNs, and hence to be a predictor of pre-malignancy [4, 18]. It has been recommended that lesions showing non-hypervascularity on dynamic imaging and hypointensity on HBP gadoxetic acid–enhanced MRI are followed for their potential to transition to HCC [19].

However, the transformation of hypovascular hypointense nodules remains controversial. A meta-analysis by Suh et al [20] of 16 studies in 944 patients with hypovascular hypointense nodules on gadoxetic acid–enhanced MRI found that the overall rate of APHE transformation was 28%, with 1-, 2-, and 3-year cumulative incidence rates of 18%, 25%, and 30%, respectively—indicating that progression to hypervascular HCC increases over time. Management of borderline DNs represents a complex situation requiring additional study.

### Western perspectives on HCC guidelines and challenges

Western guidelines on screening/surveillance, diagnosis, and management of HCC include the AASLD (2018) [21]/LiRADS (2018) [22], the EASL (2018) [23], and the Canadian Association for the Study of the Liver (CASL) (2015) [24] guidelines.

#### HCC surveillance

Surveillance can be defined as the repeated application of imaging or other modality for the detection of disease within a population at risk. The guiding principle of surveillance for HCC is to reduce overall and disease-related mortality by the early detection of HCC, at a stage when curative treatment options are possible. For this reason, surveillance has a limited role in patients with advanced or decompensated liver disease (unless they are transplant candidates), because there are no treatment options with curative intent.

The AASLD and EASL guidelines have nearly identical recommendations for surveillance, comprising US performed every 6 months [21, 23]. The AASLD guideline recognizes the additive value of measuring AFP (positive if > 20 ng/mL), while noting this may result in increased false positives and cost [21]. Patient groups at the highest risk of HCC who should receive surveillance include those with cirrhosis and/or hepatitis B viral infection. Additional study is required on the benefits of surveillance in sub-cirrhotic patients with nonalcoholic steatohepatitis (NASH; stage 2 and 3 fibrosis) or in patients with hepatitis C virus-induced advanced fibrosis or cirrhosis who have received antiviral therapy [21, 23].

The sensitivity and specificity of US, assessed in a meta-analysis of 32 surveillance studies in patients with cirrhosis, were 85% and 94%, respectively [25]. For early-stage HCC, the sensitivity of US surveillance decreased to 53%, with a specificity of 91% [25]. US therefore has low sensitivity for detection of HCC at an early stage. An additional disadvantage of US is that, in up to 20% of cases, examinations are limited by high body mass index, fatty liver, and severe cirrhosis [26]. Compliance rates for 6-monthly US surveillance are also low, reported at 34% in a large United States-based study of HCC surveillance [27].

Given the limitations of surveillance by US, researchers are investigating alternative strategies. Abbreviated MRI (AMRI) is emerging as an alternative approach, in the form of non-contrast, dynamic AMRI using extracellular contrast, or HBP AMRI using gadoxetic acid [28]. Gupta et al [29] performed a systematic review to determine the diagnostic accuracy of non-contrast and contrast-enhanced AMRI for HCC screening based on 15 studies (three prospective and 12 retrospective), including 2807 patients, 917 with HCC. The non-contrast AMRI protocol, used in 11 studies, included T1-weighted in-phase and out-of-phase imaging, T2-weighted imaging, and diffusion-weighted imaging. Pooled per-patient sensitivity and specificity of the AMRI protocols were 86% and 94%, respectively. The sensitivity and specificity of non-contrast AMRI (86% and 94%, respectively; assessed in 11 studies) were comparable to contrast-enhanced AMRI protocols (87% and 94%; 7 studies). The sensitivity of AMRI was 86% for lesions ≥ 2 cm and 69% for lesions < 2 cm. This evidence, primarily from retrospective cohorts, suggests that the sensitivity and specificity of AMRI are superior to US and can be recommended in situations where US is compromised [28].

#### HCC diagnosis

AASLD and EASL guidelines provide similar recommendations for the diagnosis of HCC based on imaging criteria [21, 23]. Recommended imaging modalities include multiphasic CT, or dynamic contrast-enhanced MRI, or (in EASL guidelines) contrast-enhanced US [21, 23]. Neither AASLD nor EASL guidelines recommend one MR contrast agent over another, although EASL guidelines note the higher sensitivity of gadoxetic acid–enhanced MRI over ECCM-MRI.

Both AASLD and EASL guidelines (in contrast to the Eastern guidelines, reported above) restrict washout on gadoxetic acid–enhanced MRI to the PVP, with the aim of retaining high specificity; hypointensity on the HBP is interpreted as an ancillary feature favoring malignancy [21, 23]. The diagnostic criteria for HCC on gadoxetic acid–enhanced MRI in Western guidelines are summarized in Table 3.

**Table 3:** Diagnostic criteria for HCC on gadoxetic acid–enhanced MRI in Western guidelines

AASLD guidelines [21]	EASL guidelines [23]
≥ 20 mm: APHE (non-rim) **AND** one or more of: • Washout (non-peripheral) • Enhancing capsule • Threshold growth 10–19 mm: APHE (non-rim) **AND** the following: • Washout (non-peripheral) • Enhancing capsule • Threshold growth	APHE plus washout (PVP only)

*Abbreviations:* *AASLD*, American Association for the Study of Liver Diseases; *APHE*, arterial phase hyperenhancement; *EASL*, European Association for the Study of the Liver; *HCC*, hepatocellular carcinoma; *MRI*, magnetic resonance imaging; *PVP*, portal venous phase

There are few head-to-head comparative studies between ECCM and gadoxetic acid, and these used variable criteria for HCC diagnosis, with mixed results depending on the diagnostic criteria used [30–37].

### Conclusions on Eastern and Western perspectives

The differences in approach between Eastern and Western countries are explainable largely by differences in the etiology, prevalence, surveillance methods, and management of HCC.

There are many challenges related to surveillance. The sensitivity of US for early-stage HCC is low, meaning that small, potentially curable HCCs are not identified [38]. Alternative strategies, such as AMRI, could potentially yield improved detection and potential survival.

In diagnosis and management, all guidelines recommend dynamic CT, dynamic MRI using ECCM, or gadoxetic acid–enhanced MRI. Guidelines do not recommend one MRI contrast agent over another, with the exception of APASL, which recommends gadoxetic acid–enhanced MRI [1], based on its higher sensitivity (particularly for the detection of small lesions). Recommendations on specific protocols for gadoxetic acid–enhanced MRI differ between Eastern and Western guidelines, reflecting differences in their requirements for specificity and sensitivity.

Gadoxetic acid-enhanced MRI provides additional information beyond HCC diagnosis, for tumor staging and prognostication, and it may have a role in identifying pre-neoplastic lesions. A reported disadvantage with gadoxetic acid is occurrences of respiratory artefacts during dynamic-phase MRI, reported at a frequency of 5–22%. While the causes and risk factors for these artifacts remain unclear, they can be mitigated by a number of optimized techniques, including shortened breath-holding times, multiple arterial phase imaging, and free-breathing acquisition [7, 39]. There is a need for more evidence on the wider applications of gadoxetic acid–enhanced MRI in HCC, in particular from clinical trials with multicenter study designs.

### Consensus statements

**Consensus statement 1:** Gadoxetic acid–enhanced MRI is useful for the diagnosis, staging, and therapy planning of HCC *(78/80 (98%) agreement).*

**Consensus statement 2:** There are regional differences in HCC diagnostic and staging systems based on population studied, available resources, and management guidelines *(81/83 (98%) agreement).*

**Consensus statement 3:** Related to the context of use and disease prevalence, Eastern diagnostic criteria favor sensitivity compared with Western diagnostic criteria that favor specificity *(77/86 (90%) agreement).*

**Consensus statement 4:** Standardized language should be used to define the adequacy of image quality for HCC diagnosis and surveillance *(79/83 (95%) agreement).*

**Consensus statement 5:** Further research is needed to establish the role of alternative surveillance strategies (including AMRI) and stratify the risk of HCC development to guide optimal surveillance strategy *(77/82 (94%) agreement).*

## Economics and outcomes in liver imaging

### Overview of cost-effectiveness analysis objectives

Cost-effectiveness analysis (CEA) is a method to inform decision-making on the economic and clinical consequences of various possible actions. CEA can be assessed by clinical trials that assess the cost in addition to clinical outcomes for a given intervention. However, such trials can be expensive, time-consuming, and—the biggest limitation—may not include downstream events or causes after the trial. Another approach, more often used in CEA, is decision-analytic modeling, which combines evidence from multiple sources: randomized controlled trials, observational methods, prospective cohort studies, case-control studies, systematic reviews, meta-analyses, and cost studies.

Decision-analytic modeling in the context of HCC diagnosis has been adopted to provide information on life expectancy, lifetime number of tests required, tests per HCC case diagnosed, and downstream costs. It should be stressed that, while CEA can provide an analysis of the consequences of each possible action, it cannot inform what is the “correct” choice.

### CEA of cross-sectional liver imaging modalities and US in HCC

#### Diagnostic imaging of HCC

Two publications compared the cost-effectiveness of gadoxetic acid–enhanced MRI versus ECCM-MRI and contrast-enhanced CT (CECT) in patients with suspected HCC. In 2017, Nishie et al [40] developed a six-stage Markov lifetime model to assess direct costs and clinical outcomes associated with each imaging modality. Diagnostic sensitivity and specificity, clinical data, treatment patterns, and costs were predominantly based on Japanese publications. Gadoxetic acid-enhanced MRI was associated with lower direct costs over a lifetime horizon (¥2,174,869 [US$19,392.50]) and generated a greater number of quality-adjusted life-years (QALYs) (9.502) than ECCM-MRI (¥2,365,421 [US$21,091.58]; 9.303 QALYs) or CECT (¥2,482,608 [US$22,136.50]; 9.215 QALYs). Most of the costs associated with HCC were treatment-related: ¥1,943,238 [US$17,327.13] for gadoxetic acid–enhanced MRI, ¥2,123,319 [US$18,932.85] for ECCM-MRI, and ¥2,212,818 [US$19,730.88] for CECT. Diagnosis of HCC using gadoxetic acid–enhanced MRI resulted in earlier detection of disease than ECCM-MRI or CECT, resulting in more effective disease management and less costly treatments.

In 2016, Lee et al [41] compared the costs associated with gadoxetic acid–enhanced MRI, ECCM-MRI, and multidetector CT (MDCT) as the initial procedure in patients with suspected HCC in South Korea and Thailand using a six-step decision-tree model. Costs were based on local costs for each diagnostic procedure or intervention. Expert consensus panels agreed that the need for further diagnostic procedures was reduced by gadoxetic acid–enhanced MRI compared to ECCM-MRI or MDCT. In South Korea, the total cost from the payer’s perspective to reach a confirmed treatment decision was US$3087/patient using gadoxetic acid–enhanced MRI, versus US$3205 for MDCT and US$3403 for ECCM-MRI. In Thailand, the total cost from the payer’s perspective was US$702 for gadoxetic acid–enhanced MRI, US$931 for MDCT, and US$873 for ECCM-MRI. Thus, the greater diagnostic certainty provided by gadoxetic acid–enhanced MRI translated to cost savings.

#### Treatment decisions in HCC

In 2018, Suh et al [42] compared the cost-effectiveness of initial workup with dynamic multiphasic CT alone or CT followed by gadoxetic acid–enhanced MRI, using a decision-analytic model for early-stage HCC. A Markov model simulated lifetime patient outcomes after curative or adjuvant treatment. The mean number of life-years gained was 7.79 with gadoxetic acid–enhanced MRI versus 7.22 with CT alone; QALY was 5.52 and 5.08, respectively, representing a modest difference. The incremental cost-effectiveness ratio (ICER) of the gadoxetic acid–enhanced MRI strategy was US$11,957 compared to CT alone, which is lower than the cost-effectiveness threshold of US$50,000/QALY. Gadoxetic acid-enhanced MRI after CT is therefore cost-effective for detecting additional HCC lesions in patients with early-stage HCC, who can undergo curative treatment.

#### HCC screening

Current guidelines recommend HCC screening by biannual US, with or without AFP assessment, in patients with cirrhosis (see Section 1, above). In 2017, in a United States–based study, Goosens et al [43] performed a cost-effectiveness assessment of screening strategies in patients with cirrhosis at high or intermediate risk of HCC. Three screening strategies were cost-effective versus biannual US: biannual full MRI or AMRI using gadoxetic acid in high- and intermediate-risk groups, or full MRI in high-risk and US in intermediate-risk groups. AMRI in high- and intermediate-risk groups had the lowest ICER of US$2100/QALY gained versus US. HCC screening in patients with intermediate- or high-risk HCC using gadoxetic acid–enhanced AMRI can therefore be cost-effective.

Vietti Violi et al [44] (2020) retrospectively assessed the screening performance of three AMRI protocols in patients with chronic liver disease at a single center in the USA. Pooled sensitivities for non-contrast AMRI (T2-weighted imaging [T2WI] + DWI), gadoxetic acid–enhanced dynamic AMRI (T2WI + DWI + dynamic T1-weighted imaging [T1WI]), and gadoxetic acid–enhanced HBP AMRI (T2WI + DWI + T1WI during HBP) were 62%, 85%, and 81%, respectively, without significant difference between the sets. Pooled specificities were 96%, 100%, and 95%, respectively, with a significant difference between dynamic AMRI and other sets (*p* < 0.01). (See also Gupta et al [29] for a comparison of non-contrast and contrast-enhanced AMRI strategies based on a meta-analysis of 15 prospective or retrospective studies, including Vietti Violi et al [44].) All AMRI methods were cost-effective compared with US, with life-year gains of 3–12 months against incremental costs of more than US$12,000. Given the higher diagnostic performance of HBP AMRI and dynamic AMRI compared with non-contrast AMRI, these were the most cost-effective models, allowing population gains of 7–12 months compared with US. Similar results were shown from a Canadian study [45].

### Nationwide health economics and outcomes research data—the South Korean perspective

#### Trends in HCC surveillance in South Korea

**Fig. 2:**
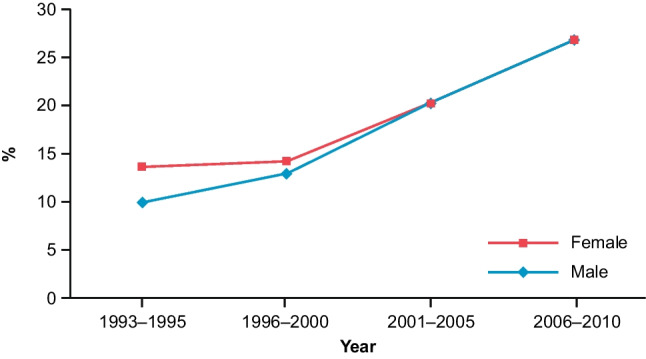
Trends in 5-year survival rates for HCC in South Korea [46]. From Yoon SK, Chun HG (2013). Status of hepatocellular carcinoma in South Korea. Chin Clin Oncol 2:39, with permission. Abbreviation: *HCC*, hepatocellular carcinoma

The 5-year overall survival rates in patients with HCC have increased rapidly in South Korea in recent years [46] (Fig. 2). This increase in survival rate can be attributed to a number of causes, including improvements in the medical management of chronic liver disease, use of antiviral treatments, and—importantly—the development of a nationwide surveillance program allowing early detection of HCC.

Biannual US and tumor marker assessment represents the current standard practice for surveillance in South Korea. However, an analysis by Kang et al at a tertiary care center showed that a large proportion of patients (88%) underwent additional CT or MRI during surveillance, suggesting that these modalities are more widely used in clinical practice than expected [47].

#### Trends in HCC diagnostic work-up in Korea

HCC is diagnosed in South Korea in three possible ways: CT alone, CT combined with gadoxetic acid–enhanced MRI, and CT combined with ECCM-MRI. To determine which is the best diagnostic option, Kang and colleagues performed a nationwide analysis on more than 30,000 patients with HCC using Korean National Health Insurance Service data [48]. After adjustment for confounders, CT plus gadoxetic acid–enhanced MRI and CT plus ECCM-MRI were associated with significantly lower all-cause mortality than CT alone (15.2, 21.7, and 36.3 deaths/100 patient-years, respectively). CT plus gadoxetic acid–enhanced MRI was associated with significantly lower mortality than CT plus ECCM-MRI in patients with localized disease, but not regional or distant disease. The cause of the differences in mortality rate is unknown, although more accurate HCC staging using gadoxetic acid–enhanced MRI than MDCT has been shown in a Korean study to improve treatment decisions [49]. The putative association of imaging modality with survival remains to be proven in randomized controlled trials.

### Conclusions on economics and outcomes in liver imaging

For HCC screening, prospective head-to-head comparisons of the performance of AMRI and US are needed. There are limited data also on the cost-effectiveness of HCC screening by etiology. Positive data are emerging on the cost-effectiveness of gadoxetic acid–enhanced MRI in HCC diagnosis. However, the relation of accurate staging to improved treatment allocation and outcome remains to be proven in randomized controlled trials. Published cost-effectiveness studies use an old willingness-to-pay threshold of US$50,000 per QALY. An updated threshold of US$100,000 per QALY could make MRI more cost-effective for liver imaging than previously documented.

### Consensus statements

**Consensus statement 6:** Emerging data on the cost-effectiveness of gadoxetic acid–enhanced MRI for HCC diagnosis are encouraging but more work is necessary *(70/83 (84%) agreement).*

**Consensus statement 7:** There are limited data on the cost-effectiveness of cross-sectional imaging including an AMRI strategy against US for HCC screening and surveillance. Thus, a prospective head-to-head comparison against US is needed *(68/82 (83%) agreement).*

**Consensus statement 8:** There are emerging data on the cost-effectiveness of MRI-based HCC screening by etiology of liver disease, particularly for NASH *(56/85 [66%] agreement, consensus not reached).*

**Consensus statement 9:** The use of MRI for HCC screening and diagnosis could be more cost-effective than previously reported. Earlier studies may no longer be applicable given the change in healthcare costs and outcomes of therapies *(64/86 (74%) agreement, consensus not reached).*

**Consensus statement 10:** The recent improvement in survival rates can be partly attributed to the early detection of HCC based on surveillance programs in high-risk populations *(68/81 (84%) agreement).*

**Consensus statement 11:** In HCC, the use of gadoxetic acid–enhanced MRI may lead to optimal treatment stratification compared with CT *(59/73 (81%) agreement).*

## Radiomics, artificial intelligence, and deep learning: current and future roles in liver imaging, including gadoxetic acid–enhanced MRI

### Clinical applications of radiomics in liver imaging

#### Principles of radiomics

Radiomics is an emerging method for the extraction of quantitative imaging features from conventional imaging modalities that are not visible to the naked eye, and correlating these features with clinical endpoints, such as pathology and therapeutic response [50, 51]. Radiomics workflow can be divided into five phases: data selection, segmentation into volumes or regions of interest, feature extraction (such as lesion size, shape, and location; histogram analysis; and texture analysis), exploratory analysis, and modeling [52].

#### Radiomics applications in liver imaging

Radiomics largely remains in the research setting, but the technique has the potential to play a pivotal role in the diagnosis, staging, and prognosis of liver disease [52]. Most effort to date has focused on liver malignancies and diffuse liver diseases.

#### Liver malignancies

##### Biologic behavior assessment

Radiomics can provide important information regarding tumor biological behavior. Cytokeratin-19 (CK-19) expression is associated with increased tumor invasion, a higher rate of lymph node metastasis, and poorer postoperative prognosis in HCC. Wang et al [53] developed a radiomics-based model derived from gadoxetic acid–enhanced MRI to preoperatively identify CK-19 status in 227 patients with a single HCC. Combining 17 radiomics features extracted from arterial-phase and HBP images, the radiomics signature achieved areas under the curve (AUCs) of 0.951 and 0.822, respectively, in training and validation datasets. A nomogram based on the final model—integrating AFP levels, arterial rim enhancement pattern, irregular tumor margin, and the fusion radiomics signature—was a reliable biomarker of CK-19 status.

Glypican 3 (GPC3) is another immunohistochemical marker, closely associated with HCC angiogenesis, invasion, metastasis, and postoperative recurrence. GPC3 is also a potential immunotherapeutic target in monoclonal antibody-based HCC therapy. Gu et al [54] identified a radiomics signature consisting of 10 features that achieved good predictive efficacy for identifying GPC3-positive HCC (training cohort: AUC, 0.879; validation cohort: AUC, 0.871). A combined nomogram integrating AFP and the radiomics signature provided AUCs of 0.926 and 0.914 in training and validation cohorts, respectively, indicating that the combined nomogram may provide a tool for individualized prediction of GPC3 positivity.

Radiomics can additionally help in predicting response to immunotherapy. Hectors et al [55] retrospectively assessed qualitative radiomics features (based on LiRADS 2018 category and ancillary findings) and quantitative features (tumor size, contrast-enhanced T1WI (CE-T1WI) enhancement ratios, and ADC analysis) for the prediction of immuno-oncologic characteristics in 48 patients with HCC. Qualitative and quantitative features correlated with immunohistochemical cell type markers for T-cells (CD3), macrophages (CD68), and endothelial cells (CD31). Radiomics features also correlated with the expression of immunotherapy targets: programmed cell death ligand 1 at the protein level and programmed cell death protein 1 (PD-1) and cytotoxic T-lymphocyte–associated protein 4 at the RNA expression level. Finally, radiomics features showed significant performance in the assessment of early HCC recurrence (AUC, 0.76–0.80; *p* < 0.043).

##### Prognosis prediction

Several studies have investigated radiomics findings related to patient outcomes after different therapies. Ji et al [56] assessed a pre-operative model that integrated a CT radiomics signature (based on 20 features) with AFP and the number of tumors, and a postoperative model that incorporated microvascular invasion and satellite nodules into the predictors, in 295 patients undergoing resection for early HCC. The two radiomics-based models provided better predictive performance for recurrence-free survival and lower prediction error than alternative models without radiomics or other widely adopted staging systems. In addition, the radiomics-based models gave three risk strata with high, intermediate, or low risk of recurrence and distinct profiles of recurrent tumor number.

#### Challenges and future perspectives of radiomics

 Despite promising results in liver imaging research, radiomics analysis remains a young discipline and applications in HCC are relatively limited. Most studies to date have been retrospectively designed, with the potential for substantial selection bias in the patient populations. Radiomics features are also highly dependent on the protocols for imaging, segmentation, and feature extraction. Lack of standardization in imaging acquisition parameters and segmentation methods also negatively impacts reproducibility and comparability between studies.

Despite these challenges, the potential of radiomics is high in each phase of HCC management. Well-designed, large-scale, multicenter prospective studies should be encouraged to verify preliminary results. Efforts should also be made to standardize acquisition, segmentation, and post-imaging processing: for example, using open-source tools such as GitHub, PyRadiomics, or MATLAB, to enable the development and validation of radiomics analysis across multiple institutions. Combining radiomics with AI holds the promise of benefiting from the advantages of both techniques.

Another important aspect in the development of radiomics will be to qualify this highly promising tool as a non-invasive surrogate capable of reflecting the complex biologic heterogeneity of HCC and the surrounding micro-environment both in primary and metastatic disease [57, 58], an effort that will require integration with parallel tissue phenotyping.

### Use of AI for image reconstruction and liver lesion characterization

AI applications have an important role in image reconstruction by improving image quality and reducing noise, and may in the future make important contributions to lesion detection, characterization, and the assessment of treatment response [59].

#### AI reconstruction

**Fig. 3:**
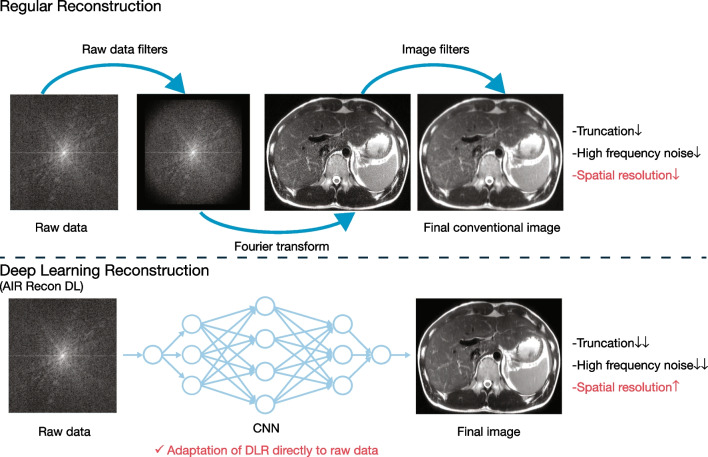
DL reconstruction. *Abbreviations: CNN*, convolutional neural network; *DL*, deep learning; *DLR*, deep learning reconstruction; *ITK*, insight toolkit. Courtesy GE Healthcare Japan

AI reconstruction algorithms use deep convolutional neural networks to reduce noise and improve spatial, contrast, and temporal resolution in CT or MR images. An example of a DL-reconstruction (DLR) algorithm is shown in Fig. 3, which compares regular reconstruction (upper images) with DLR (lower images). DLR can reconstruct images without k-space filters or Fourier transformation; instead, this technique adapts a DL network directly to the raw data to reconstruct images. Compared to regular reconstruction, truncation artifacts and high-frequency noise can be substantially reduced and spatial resolution improved.

#### AI imaging for tumor detection, characterization, and assessment of treatment response

Most patients with HCC undergo multiple imaging studies: screening by US; diagnosis by contrast-enhanced MRI or CT; and follow-up imaging post treatment. Each imaging study acquires numerous data points that may be used for image post processing and analysis. The vision for AI is to apply machine learning algorithms to these multi-parametric data to provide fully automated, fast, reproducible, and reliable tumor detection and characterization and clinical decision support.

**Fig. 4:**
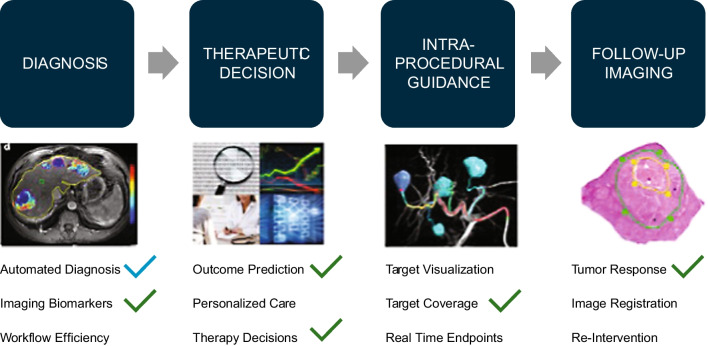
How advanced data analysis can help us. Courtesy J Chapiro

Figure 4 summarizes the investigations to date by different research groups. Investigators have examined automation of diagnosis and use of imaging biomarkers, prediction of outcome in personalized care and therapy decision-making, targeted coverage, and tumor response. However, other areas remain to be investigated, as discussed below.

##### Automation of tumor detection

The LiRADS criteria have been developed to improve radiologic diagnosis by reducing variability in interpretation, but the increasing complexity of LiRADS is hindering its implementation in high-volume clinical practice [60].

Bousabarah et al [61] performed a feasibility study to establish the proof-of-principle of automating the detection and segmentation of HCC lesions, using a DL algorithm trained to automatically segment the liver and delineate HCCs on MRI. Multiphasic contrast-enhanced MRIs using T1W sequences acquired on 174 patients from 2010 to 2018 were used to train a deep convolutional neural network (DCNN). The dice similarity coefficient (DSC) was measured between manual and DCCN methods. Post-processing using a random forest (RF) classifier employing radiomics features and thresholding (TR) of the mean neural activation reduced the average false-positive rate. The mean DSC between automatically detected lesions using the DCNN + RF + TR and corresponding manual segmentations was 0.64/0.68 (validation/test), and 0.91/0.91 for liver segmentations. The DCNN therefore has a high level of performance in the delineating liver and focal liver lesions and could enable a more efficient workflow.

##### Automation of diagnosis

Hamm et al [60] reported a proof-of-concept study of a CNN-based DL system (DLS) that classified common liver lesions using multiphasic contrast-enhanced MRI. Augmentation techniques generated 43,400 training samples depicting 494 hepatic lesions: simple cysts, cavernous hemangiomas, focal nodule hyperplasia, HCCs, intrahepatic cholangiocarcinomas, and colorectal cancer metastases. In the test set (*n* = 60), DLS demonstrated 92% accuracy, 92% sensitivity, and 98% specificity. The sensitivity of the DLS was 90% for classifying HCC, compared to 60%/70% for two radiologists with ≥ 20 years’ total experience. With a computation time to classify each lesion of 5.6 msec, CNN could help make the clinical workflow more efficient.

Professor Murakami and colleagues are developing an AI diagnostic system for detecting HCC based on gadoxetic acid–enhanced MRI. This machine-learning technique distinguishes cancerous and non-cancerous areas by calculating the average and distribution of signal intensity in images from each conventional MR sequence after registration of the relative position coordinates of the images in a 3D location, which enables the detection and characterization of small focal liver lesions. The AI tool aims to support the interpretation of gadoxetic acid–enhanced MRI by reducing the number of missed/overlooked small lesions.

##### Radiologic-pathologic validation of the AI classification result

The next step is radiologic-pathologic validation of the classification results. Oestmann et al [62] sought to train a DL model to differentiate pathologically proven HCC and non-HCC lesions, including lesions with atypical imaging features on MRI, based on a retrospective study in 118 patients. A 3D CNN was trained on 140 lesions and tested for its ability to classify the 10 remaining lesions. CNN predicted histopathologic diagnosis with a high level of accuracy (87%) with a computational time < 3 msec, potentially reducing the need for biopsy in atypical HCC.

##### DL explainability—why DL does what it does

Although AI could enhance clinical workflow in diagnosis, prognosis, and treatment, transparency is a vital component that clinicians will require before acceptance. How do we take AI decisions out of the ‘black box’, and how do we provide an interpretable DLS for a liver tumor diagnosis?

Wang et al [63] developed a DL prototype that justifies aspects of its predictions. Fourteen radiologic features were selected comprising characteristics observable on multiphasic MRI, and subsets of lesions with these features were passed through a CNN that was engineered and trained to classify six hepatic tumor entities. The CNN system’s performance was assessed by its ability to identify a test set of 60 lesions. The interpretable DL network achieved 76.5% positive predictive value and 83.9% sensitivity in correctly identifying the radiologic features.

##### Can neural networks help predict response to therapy before we treat?

Investigators have attempted to integrate clinical information and baseline imaging features into an artificial neural network, with the output of a prediction of whether a patient will be a TACE responder or a non-responder [64]. Clinical information (presence of cirrhosis), baseline imaging (pre-TACE tumor signal intensity > 27 relative intensity units, number of tumors > 2), and therapeutic features (received conventional TACE, previously or simultaneously treated with sorafenib) were used to train logistic regression (LR) and RF models. LR and RF models predicted TACE treatment response with an accuracy of 72% and 66%, respectively. The highest overall accuracy (78%) was achieved when the models were trained with two features: pre-TACE imaging signal intensity > 27, and presence of cirrhosis.

## Conclusions

At present, DL algorithms have been implemented in CT and MRI, where they can improve image quality and shorten scanning time. In the future, AI and radiomics will contribute together in the detection, staging, assessment of treatment response, and prediction of prognosis—thereby reducing the burden on doctors.

Transparency is a vital component that clinicians will require before acceptance. Explainable AI is under development to help understand and interpret the predictions made by machine-learning models. The development of explainable AI is highly desirable for the practical applications of AI to progress. Another major need for clinical translation is the availability of well-curated, diverse, and reliable training data, potentially achieved using federated learning to share data across institutions.

### Consensus statements

**Consensus statement 12:** Data-driven learning may provide insight into tumor biology prognosis and treatment response, which may help to develop novel biomarkers for screening, surveillance, lesion characterization, and patient treatment allocation *(82/86 (95%) agreement).*

**Consensus statement 13:** DL-based reconstruction for cross-sectional imaging may reduce noise and improve image quality with the potential to decrease acquisition time *(67/76 (88%) agreement).*

**Consensus statement 14:** There is an unmet need for standardized and unified data formats, quality control, annotation, and feature quantification, using data-driven learning in multi-institutional data *(70/81 (86%) agreement).*

**Consensus statement 15:** The lack of well-curated, publicly available datasets limits the validation and translation of data-driven learning methodologies towards effective clinical applications *(63/78 (81%) agreement).*

**Consensus statement 16:** Federated learning and other emerging technologies may facilitate translation from code to bedside while minimizing ethical and privacy dilemmas in multi-institutional data projects *(52/82 (63%) agreement, consensus not reached).*

## Summary

Delegates at the 10th Global Liver Forum discussed differences in the protocols for gadoxetic acid–enhanced MRI between Eastern and Western guidelines, evidence on the cost-effectiveness of gadoxetic acid–enhanced MRI in HCC screening and diagnosis, and the potential roles of AI and radiomics in the detection, staging, assessment of treatment response, and prediction of prognosis of HCC in clinical practice.

### Supplementary Information

Below is the link to the electronic supplementary material.

Supplementary file1 (PDF 875 KB)

## Abbreviations

AASLD: American Association for the Study of Liver Diseases
AFP: Alfa-fetoprotein
AI: Artificial intelligence
AMRI: Abbreviated MRI
APASL: Asia-Pacific Association for the Study of the Liver
APHE: Arterial phase hyperenhancement
CASL: Canadian Association for the Study of the Liver
CEA: Cost-effectiveness analysis
CECT: Contrast-enhanced computed tomography
CK-19: Cytokeratin-19
CNLC: Chinese Liver Cancer
CNN: Convolutional neural network
DCNN: Deep convolutional neural network
DL: Deep learning
DLR: Deep learning reconstruction
DLS: Deep learning system
DN: Dysplastic nodule
DSC: Dice similarity coefficient
EASL: European Association for the Study of the Liver
ECCM: Extracellular contrast media
ECCM-MRI: Extracellular contrast media-magnetic resonance imaging
GPC3: Glypican 3
HBP: Hepatobiliary phase
ICER: Incremental cost-effectiveness ratio
JSH: Japan Society of Hepatology
KLCA-NCC: Korean Liver Cancer Association-National Cancer Center Korea
NASH: Nonalcoholic steatohepatitis
PD-1: Programmed cell death protein 1
PVP: Portal venous phase
QALY: Quality-adjusted life-year
RF: Random forest
RFA: Radiofrequency ablation
T1WI: T1-weighted imaging
T2WI: T2-weighted imaging
TACE: Transarterial chemoembolization
TP: Transitional phase

## Declarations

### Guarantor

The scientific guarantor of this publication is Bachir Taouli, MD, MHA.

### Statistics and biometry

No complex statistical methods were necessary for this paper.

### Study subjects or cohorts overlap

Not applicable as this is a review paper.

### Methodology

- Not applicable as this is a review paper.
