# Supplementary material for: Consensus report from the 10th Global Forum for Liver Magnetic Resonance Imaging: developments in HCC management
Source: Eur Radiol. 2023 Jul 27;33(12):9152–66. doi: 10.1007/s00330-023-09928-y (PMC10730664; doi:10.1007/s00330-023-09928-y)
Supplement: Supplementary file 1 — Supplementary file1 (PDF 875 KB) [file 330_2023_9928_MOESM1_ESM.pdf]

Consensus report from the 10th Global Forum for Liver Magnetic Resonance Imaging: developments in HCC management

Electronic Supplementary Material

Eur Radiol (2023) Taouli B, Ba-Ssalamah A, Chapiro J et al.

Questionnaire survey results: global survey on gadoxetic acid usage

A survey developed by Professor Taouli and a Bayer research team was completed by 453 radiologists from China, Germany, Italy, Japan, South Korea, and USA in advance of the 10th Global Liver Forum to explore current practice in liver MRI. Professor Taouli presented and commented on the survey results at the Forum.

Survey methodology

The survey was a quantitative, double-blinded 25-minute online questionnaire performed between August 2021 and December 2021 by specialized/board radiologists who had been certified for at least 3 years and fewer than 35 years. The participants were from China (*n* = 101), Germany (*n* = 80), Italy (*n* = 52), Japan (*n* = 70), South Korea (*n* = 50), and USA (*n* = 110).

Referrals for imaging

Referrals for liver imaging (CT or MRI) came almost equally from abdominal/liver surgeons, oncologists, hepatologists, and gastroenterologists, with the remainder from internal medicine physicians, general practitioners (GP)/primary care physicians (PCP), and other.

Referrals for liver imaging come almost equally from surgeons, oncologists, hepatologists, and gastroenterologists

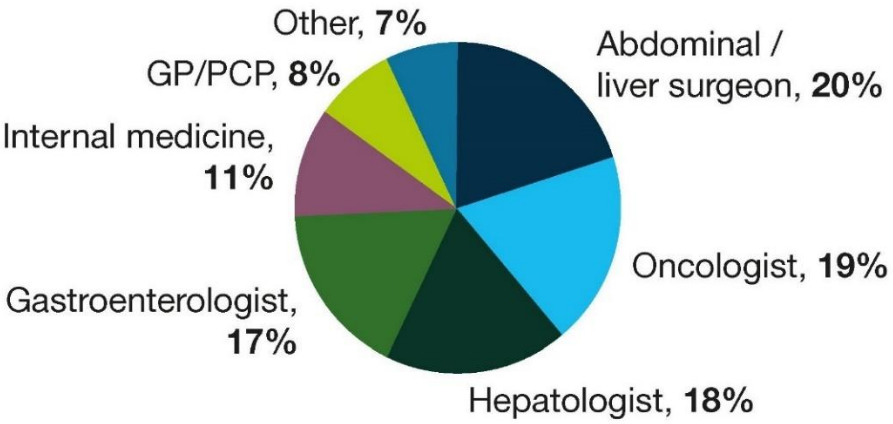

The most common reasons for referrals for liver MRI were indeterminate lesions on US or CT, HCC screening/surveillance, HCC diagnosis/staging, liver metastases diagnosis and staging, and diseases of the biliary system or pancreatic disease.

Indications for liver MRI

Most frequent: indeterminate lesion on US/CT

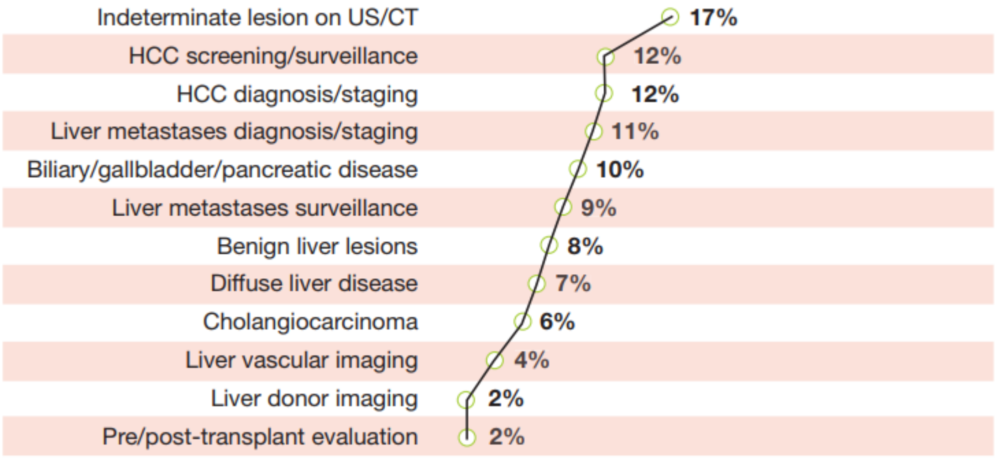

MRI contrast agent choice

In the majority of countries, with some variation, the radiologist primarily decides which contrast agent is injected into the patient: up to 99% in Germany, 9l% in USA, and slightly less in South Korea, where there is also a decision made by the referring physician.

Decisions regarding choice of contrast agent largely lie with radiologists

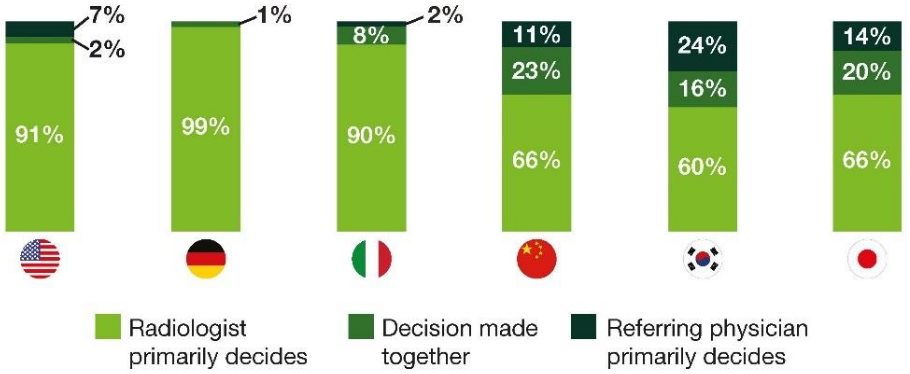

Liver imaging practice

In most countries, CT is still the predominant liver imaging modality, as expected: from 69% in South Korea to as low as 39% in Italy, but ~40–70% of cases overall.

For MRI, there are a large proportion of images with ECCM and a smaller proportion (about 19–34%) with gadoxetic acid-MRI. Data from a 2016 survey of radiologists in Germany, Italy, and USA show there has been minimal change in the use of gadoxetic acid.

Liver imaging practice differs by country

Korea/Japan: high use of CT and a preference for gadoxetic acid for MRI

2016 data (gadoxetic acid-MRI)

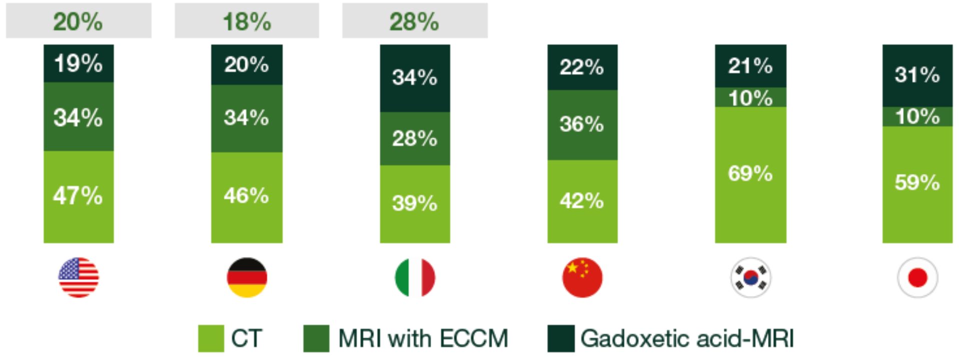

Focusing on MRI

In Asia, most MRIs for liver indications are done with gadoxetic acid (75% in Japan, 67% in South Korea). About one third of liver MRIs in Germany and USA are with gadoxetic acid, which increases to 55% in Italy.

Liver MRI primarily performed with ECCM in USA, Germany, China
High proportion of gadoxetic acid in Korea/Japan

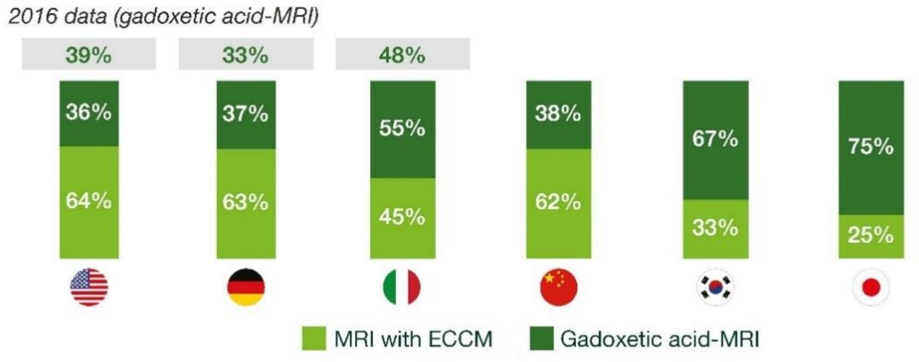

Note: Data is not weighted by patient case load.

Institutional policies

A majority of institutions have a policy or standard operating procedure (SOP) in place for MRI contrast use—up to 72% in South Korea. By contrast, overall, about 20–25% of institutions do not have a policy in place.

Internal policy/SOP in place for MRI contrast use

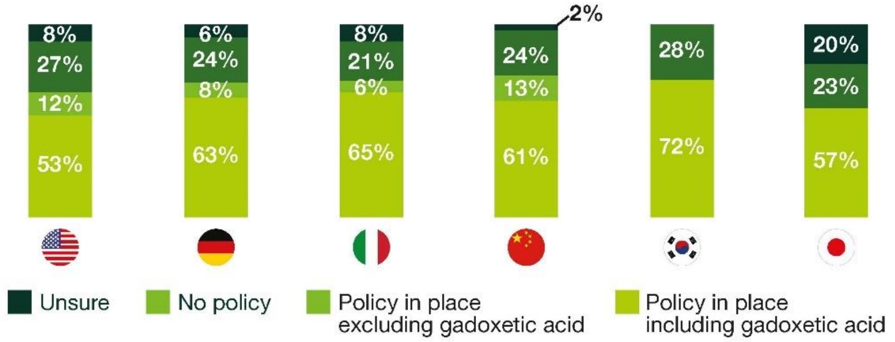

Gadoxetic acid use in different clinical scenarios

The distribution of use of CT, MRI with extracellular contrast, and MRI with gadoxetic acid is almost even for indeterminate lesions, HCC diagnosis and staging, and screening and surveillance (all regions combined).

Gadoxetic acid is used across different clinical scenarios

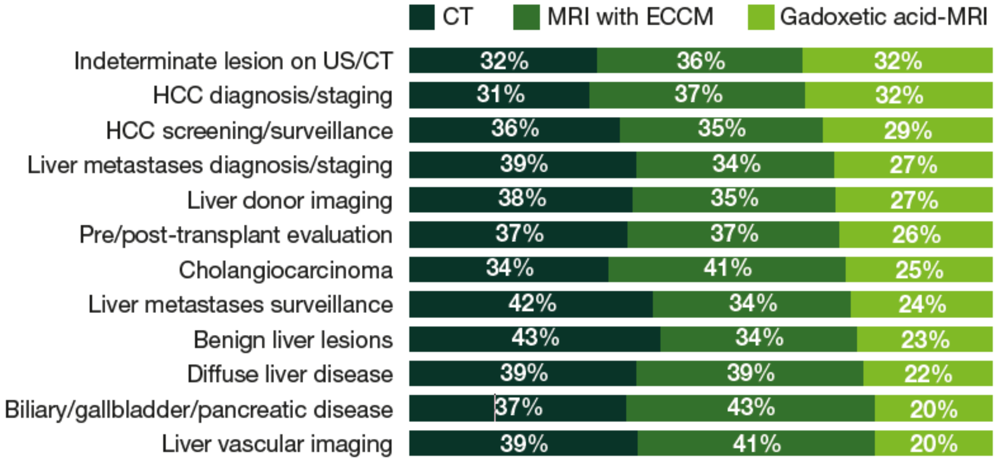

<table>
<tr>
<td>Perceived value of gadoxetic acid-enhanced MRI
Gadoxetic-enhanced MRI was perceived as valuable or very valuable for problem solving, treatment planning, and treatment follow-up—whether for HCC or metastases.</td>
<td>Gadoxetic acid-MRI is most valuable for problem solving
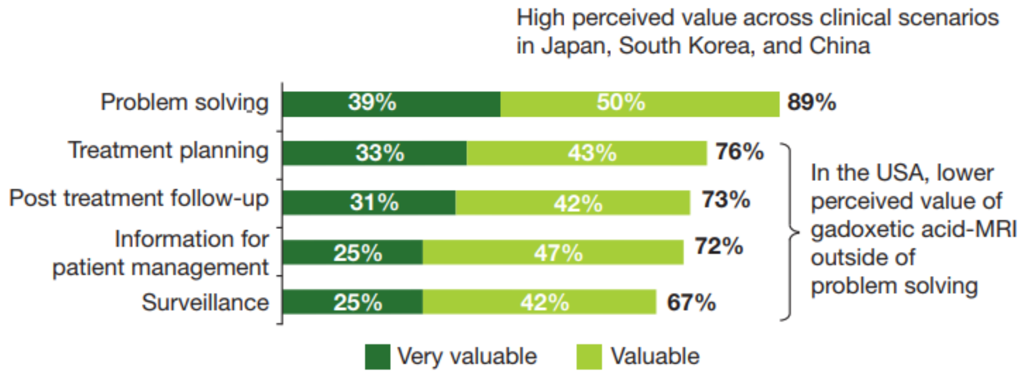

</td>
</tr>
<tr>
<td>Barriers to gadoxetic acid use
The top reason for not using gadoxetic acid was cost, which applied to most countries. Radiologists were also satisfied with extracellular agents, which were incorporated into standardized reporting protocols. Another issue was arterial phase imaging with gadoxetic acid. Finally, there were other issues with workflow, difficulty interpreting washout, etc.</td>
<td>Barriers for gadoxetic acid use
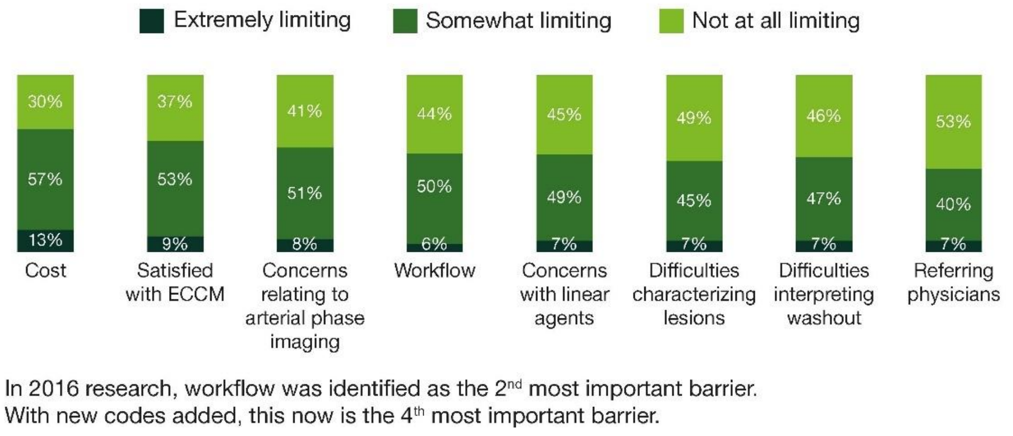

</td>
</tr>
</table>

Survey results showed that the LiRADS group has gained widespread acceptance—globally around 78%. In Japan, 90% of surveyed radiologists were using local guidelines from the Japanese Society of Hepatology and only 32% were using LiRADS. Interestingly, the United States Organ Procurement and Transplantation Network (OPTN) guidance was used by 20% of surveyed radiologists in China.

LiRADS is the most commonly used classification system for HCC (except Japan/Korea)

% radiologists using each of the following classification systems	LiRADS	OPTN	EASL	APASL	JSH	Korean Guidelines
Global	78%	11%	24%	22%	14%	5%
🇺🇸	95%	7%	4%	0%		
🇩🇪	63%	4%	35%	3%		
🇮🇹	71%	4%	44%	8%		
🇨🇳	87%	20%	45%	48%		
🇰🇷	66%	0%	0%	2%		98%
🇯🇵	33%	3%	7%	7%	90%	

Gadoxetic acid dosing

There was variation in the dosing used: some radiologists used a fixed dose of 10 mL (related to the desire to avoid waste), but ~55–93% of surveyed radiologists were using a Food and Drug Administration- or European Medicines Agency-approved dose of 0.1 mL/kg (radiologists from Japan and USA were not surveyed).

Weight-based dosing is most common

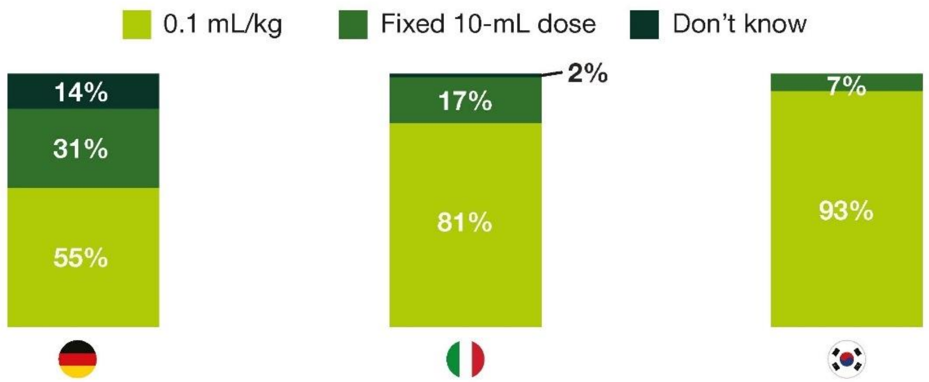

<table>
<tr>
<td>

Protocol for arterial phase imaging

The protocol used for arterial phase imaging with gadoxetic acid also showed regional variation. The top three methods were: fixed delay, a test bolus for arterial phase timing, or fluoroscopic triggering/automated bolus detection algorithm.

</td>
<td>

Arterial phase timing techniques for gadoxetic acid

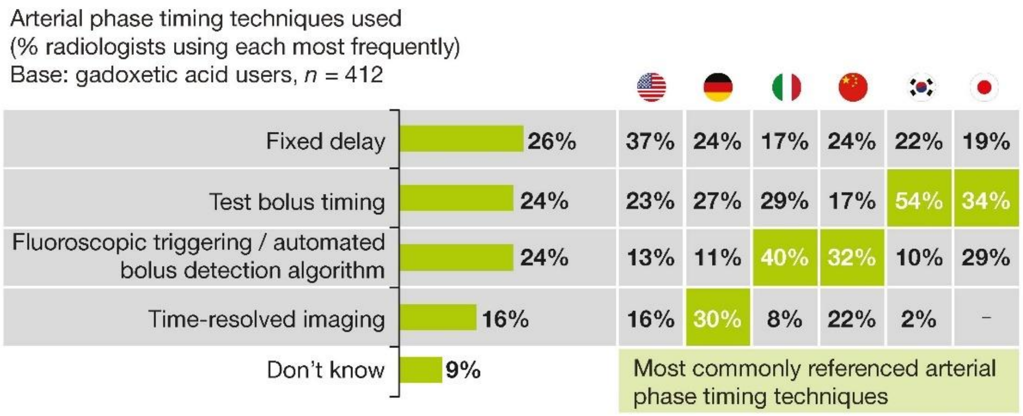

</td>
</tr>
<tr>
<td>

Vendors used by the radiologists

Globally, about half the surveyed radiologists were using a Siemens scanner, followed by GE, then Philips. Field strength was basically equal between 3T and 1.5T.

</td>
<td>

Scanner vendors and field strengths for gadoxetic acid-MRI

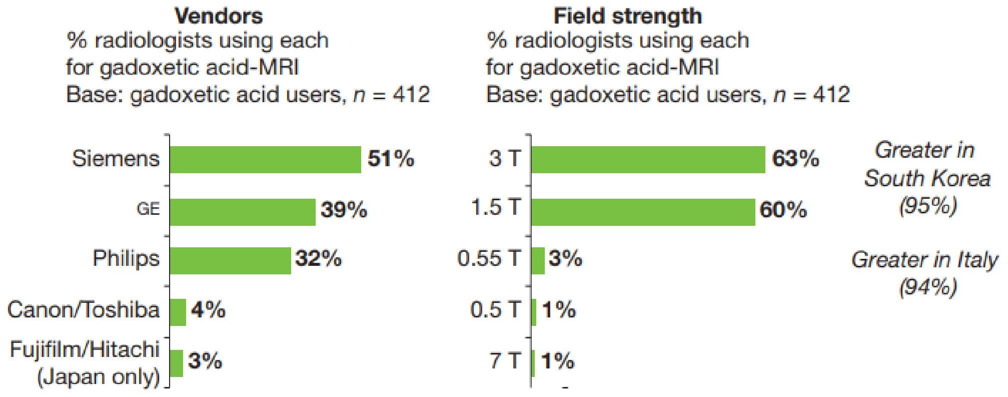

</td>
</tr>
<tr>
<td>

Abbreviated protocols with gadoxetic acid

Globally, approximately one third of surveyed radiologists were using some form of abbreviated protocol: from 39% in China (the highest percentage) to 20% in Japan.

</td>
<td>

Globally, ~1/3 are using abbreviated protocols

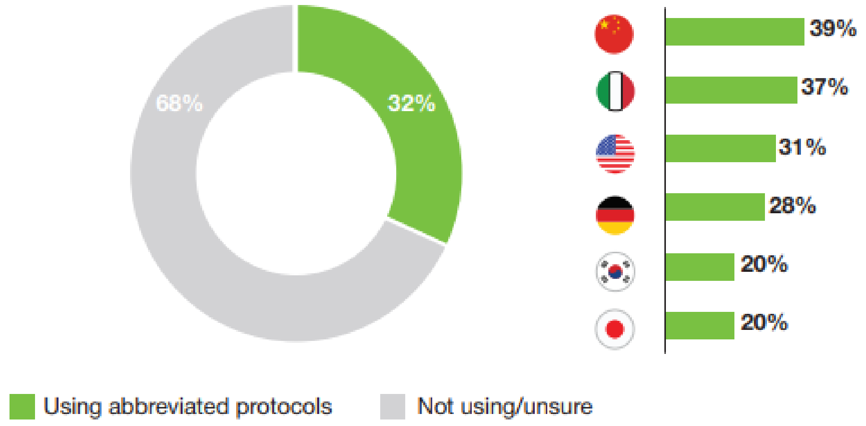

</td>
</tr>
</table>

The scenarios for using abbreviated protocols with gadoxetic acid varied. The top indication was initial diagnosis of liver metastases, which is an interesting finding. We assume that HCC diagnosis is an additional use of the abbreviated protocol, but we did not ask that question.

Abbreviated protocols are used across different clinical scenarios

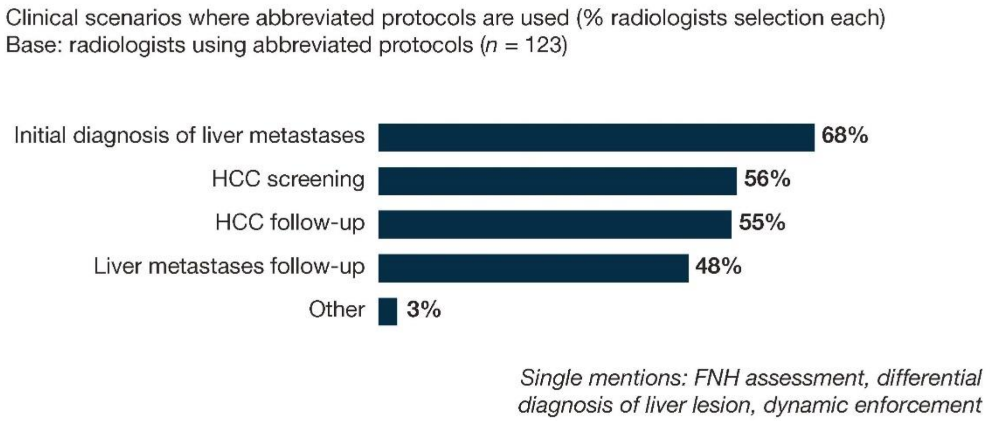

Perception of abbreviated protocols

84% of surveyed radiologists considered that abbreviated MRI protocols were better or significantly better than US for HCC screening and surveillance.

Perception of abbreviated MRI protocols: perform better than US for HCC screening

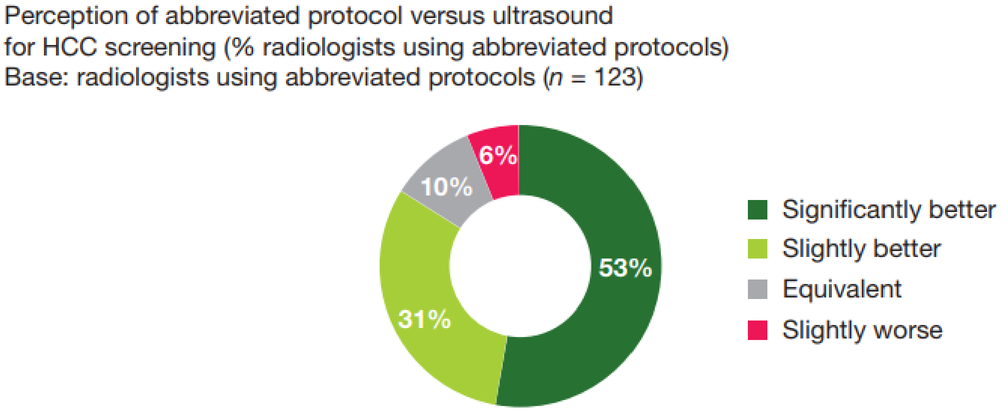

Summary of survey outcomes

- Gadoxetic acid versus ECCM use is stable/minimally increased in USA/Europe.
- Gadoxetic acid is used in 19–34% of cases.
- Gadoxetic acid is used more often than ECCM in Asia.
- Most radiologists viewed gadoxetic acid as a problem-solving agent.
- Most radiology centers have contrast guidelines that reference gadoxetic acid.
- Barriers to gadoxetic acid use are cost and satisfaction with ECCM.
- There is a potential role for abbreviated MRI using gadoxetic acid.
